# Moderate alcohol consumption is associated with lower chronic disease burden expressed in disability-adjusted life years: a prospective cohort study

**DOI:** 10.1007/s10654-017-0247-x

**Published:** 2017-04-13

**Authors:** Joline W. J. Beulens, Heidi P. Fransen, Ellen A. Struijk, Jolanda M. A. Boer, G. Ardine de Wit, N. Charlotte Onland-Moret, Jeljer Hoekstra, H. Bas Bueno-de-Mesquita, Petra H. M. Peeters, Anne M. May

**Affiliations:** 1 Julius Center for Health Sciences and Primary Care, University Medical Center Utrecht, PO Box 85500, 3508 GA Utrecht, The Netherlands; 2 Department of Epidemiology and Biostatistics, EMGO + Institute for Health and Care Research, VU University Medical Center, Amsterdam, The Netherlands; 3 National Institute for Public Health and the Environment (RIVM), Bilthoven, The Netherlands; 4 Department of Gastroenterology and Hepatology, University Medical Center, Utrecht, The Netherlands; 5 Department of Epidemiology and Biostatistics, The School of Public Health, Imperial College London, London, UK; 6 Department of Social and Preventive Medicine, Faculty of Medicine, University of Malaya, Kuala Lumpur, Malaysia

**Keywords:** Alcohol consumption, Disability-adjusted life years, Chronic disease burden, Cancer, Cardiovascular disease

## Abstract

The relation of alcohol consumption with disease burden remains debated partly due to opposite associations with cardiovascular disease (CVD) and cancer. The relation of alcohol consumption with disease burden expressed in disability-adjusted life years (DALYs) summarizes opposing associations of alcohol consumption on chronic diseases. This study aimed to investigate the association of alcohol consumption with chronic disease burden expressed in DALYs based on individual-participant data. The study was a prospective study among 33,066 men and women from the EPIC-NL cohort. At baseline, alcohol consumption was assessed with a validated food-frequency questionnaire. Participants were followed for occurrence of and mortality from chronic diseases and DALYs were calculated. After 12.4 years follow-up, 6647 disease incidences and 1482 deaths were documented, resulting in 68,225 healthy years of life lost (6225 DALYs). Moderate drinkers (women 5–14.9 g/day, men 5–29.9 g/day) had a lower chronic disease burden (mean DALYs −0.27; 95% CI −0.43; −0.11) than light drinkers (0–4.9 g/day), driven by a lower disease burden due to CVD (−0.18: −0.29; −0.06) but not cancer (−0.05: −0.16; 0.06). The associations were most pronounced among older participants (≥50 years; −0.32; −0.53; −0.10) and not observed among younger women (−0.08; −0.43; 0.35), albeit non-significant (p_interaction_ > 0.14). Substantial drinking (women 15–29.9 g/day, men 30–59.9 g/day) compared to light drinking was not associated with chronic disease burden. Our results show that moderate compared to light alcohol consumption was associated with living approximately 3 months longer in good health. These results were mainly observed among older participants and not seen among younger women.

**Electronic supplementary material:**

The online version of this article (doi:10.1007/s10654-017-0247-x) contains supplementary material, which is available to authorized users.

## Introduction

Moderate alcohol consumption, 1–2 drinks per day [1–4], has been associated with a reduced risk of all-cause mortality in prospective cohort studies compared to abstainers and heavier drinkers [5]. This reduced risk is ascribed to a reduced risk of cardiovascular diseases with moderate alcohol consumption [6, 7], but remains disputed [8]. Conversely, any amount of alcohol consumption increases the risk of different types of cancer [9, 10]. Alcohol consumption is linearly associated with an approximately 10% increased risk of breast cancer with each drink per day [9]. These opposing associations of alcohol consumption with different chronic diseases emphasizes the importance of investigating the relation of alcohol consumption with total disease burden, which can be estimated with disability-adjusted life years (DALYs).

To date, DALYs have mainly been calculated on a population level based on surveillance data of disease incidence and mortality, like in the Global Burden of Disease Study (GBD) [11]. In those studies, risk factors such as alcohol consumption are related to DALYs based on effect sizes from observational or intervention studies. According to the GBD of 2010, alcohol use accounted for up to 5% of the global disease burden, but this estimate differed over different populations and regions [11]. However, the GBD studies provides a population estimate that may be influenced by different drinking patterns of populations. For example, the burden of alcohol use was greater among men than women and particularly high in certain regions such as Eastern Europe [11]. This can be explained by women drinking less on average than men. In addition, the higher contribution of injury due to alcohol use stronger affects men than women but also explains higher disease burden in certain regions like Eastern Europe [12–15].

We previously calculated DALYs at an individual level for participants of the EPIC-NL cohort who were followed for disease occurrence to study the relation between lifestyle factors and disease burden [16, 17]. We now use this approach to account for opposing associations of alcohol consumption with chronic diseases, including the influence of dosage and adjustment for confounding. We will also address effect modification by sex and age.

## Methods

### EPIC-NL study

The EPIC-NL study consists of the two Dutch contributions to the European Prospective Investigation into Cancer and Nutrition (EPIC), which were set up simultaneously between 1993 and 1997 [18]. In brief, the Prospect-EPIC study includes 17,357 women (49–70 years) who participated in the Dutch breast cancer screening programme. The MORGEN-EPIC cohort consists of 22,654 men and women (20–65 years) from random samples of the population in three Dutch towns (Doetinchem, Amsterdam and Maastricht). At baseline, a general questionnaire and a food-frequency questionnaire (FFQ) were administered and a physical examination was performed. This study was conducted according to the Declaration of Helsinki and approved by the local institutional review boards. Written informed consent was obtained from all subjects.

From the total cohort (n = 40,011), participants without permission for linkage with disease registries were excluded (n = 2879). Participants with any of the studied diseases at baseline [Cancer, Coronary Heart Disease (CHD), Stroke, Diabetes Mellitus, Chronic Obstructive Pulmonary Disease (COPD), Asthma, Parkinson’s disease, Rheumatoid Arthritis, Osteoarthritis, and Inflammatory Bowel Disease (IBD)] (n = 3625) were excluded. We also excluded subjects with missing information from the FFQ on alcohol consumption and dietary intake (n = 142) or with implausible high or low scores for total energy intake (those in the top 0.5% and bottom 0.5% of the ratio of reported energy intake over estimated energy requirement based on basal metabolic rate) (n = 299), leaving 33,066 men and women for analysis.

### Alcohol consumption

Alcohol consumption was assessed by the general questionnaire and a food frequency questionnaire (FFQ). The FFQ contained questions on the usual frequency of consumption of 79 main food items during the year preceding enrolment [19, 20]. This questionnaire allowed the estimation of the average daily consumption of 178 foods, including alcoholic beverages. The relative validity of alcohol consumption measured with the FFQ is good as confirmed by a Spearman correlation of 0.87 between the FFQ and the average of 12 monthly 24-h recalls [19, 20]. Subjects were asked whether they formerly or currently used alcohol. If doing so currently, they were asked the number of units of beer, white wine, red wine, port/sherry/vermouth and spirits consumed. Subjects indicated their consumption frequency on a daily/weekly/monthly/yearly scale or as never consumed. Alcohol consumption was determined based on the FFQ by multiplying the amount of each beverage consumed by their alcohol content using the standard ethanol weight content (5% for beer, 18.5% for fortified wine, 12.5% for red wine, 12% for white wine and 40% for liquor; means from the average type of beverages) and was categorized into 5 categories of total alcohol consumption; lifetime abstainers, former drinkers, light drinkers (0–4.9 g/day), moderate drinkers (5–14.9 g/day for women, 5–29.9 g/day for men), and substantial drinkers (≥15 g/day for women and ≥30 g/day for men). Substantial drinkers were further subdivided into heavy drinkers (15–29.9 g/day for women, 30–59.9 g/day for men) and excessive drinkers (≥30 for women, ≥60 for men).

### Covariates

The general questionnaire included questions on presence of risk factors for chronic diseases, like smoking status and intensity (categorized as never; former: quit smoking >20 years ago, quit 10–20 years ago, quit ≤10 years ago; current smoker 1–15 cigarettes/day, 16–25 cigarettes/day, >25 cigarettes/day; pipe or cigar smoker) and level of education [categorized as very low (only primary school), low (lower vocational education), middle (secondary school or intermediate vocational training) or high (higher vocational training or university)]. Usual dietary intake was obtained from the validated FFQ and was used to score intake of eight components of a Mediterranean-style Diet: vegetables; legumes; fruit, nuts and seeds; cereals; fish; the ratio of unsaturated to saturated fatty acids; meat; and dairy products [21]. Physical activity was assessed using the EPIC physical activity questionnaire and categorized according to the validated Cambridge Physical Activity Index (CPAI) [22, 23] in 4 categories (inactive, moderately inactive, moderately active or active). Missing data on physical activity (14%), smoking status (0.4%), educational level (0.8%), and/or BMI (0.1%) was imputed using single linear regression modelling (SPSS MVA procedure).

### Endpoint assessment

Participants were followed for mortality and morbidity through linkage with several registries. The selection of the diseases was based on their prevalence and disease burden in the Netherlands, but also on the availability of data sources. Information on vital status and the date of death was obtained through linkage with municipal registries. Causes of death were obtained from Statistics Netherlands. Information on disease occurrence (Cancer, CHD, Stroke, Diabetes Mellitus, COPD, Asthma, Parkinson’s disease, Rheumatoid Arthritis, Osteoarthritis, and IBD) was obtained from the National Cancer Registry and the national hospital discharge diagnosis database from the Dutch National Medical Registry. The National Cancer Registry provided information on the type of cancer and the date of histological diagnosis. The national hospital discharge diagnosis database provided the date of diagnosis for CHD, stroke, Diabetes Mellitus, COPD, Asthma, Parkinson’s disease, Rheumatoid Arthritis, Osteoarthritis, and IBD. The national hospital discharge diagnosis database was linked to the cohort with a validated probabilistic method [24]. Self-report and urinary glucose strip tests provided additional information on diabetes. Diabetes cases were verified against information of the general practitioner or pharmacist [25]. Follow-up was complete until 31 December 2007.

### Computation of DALYs

**Fig. 1:**
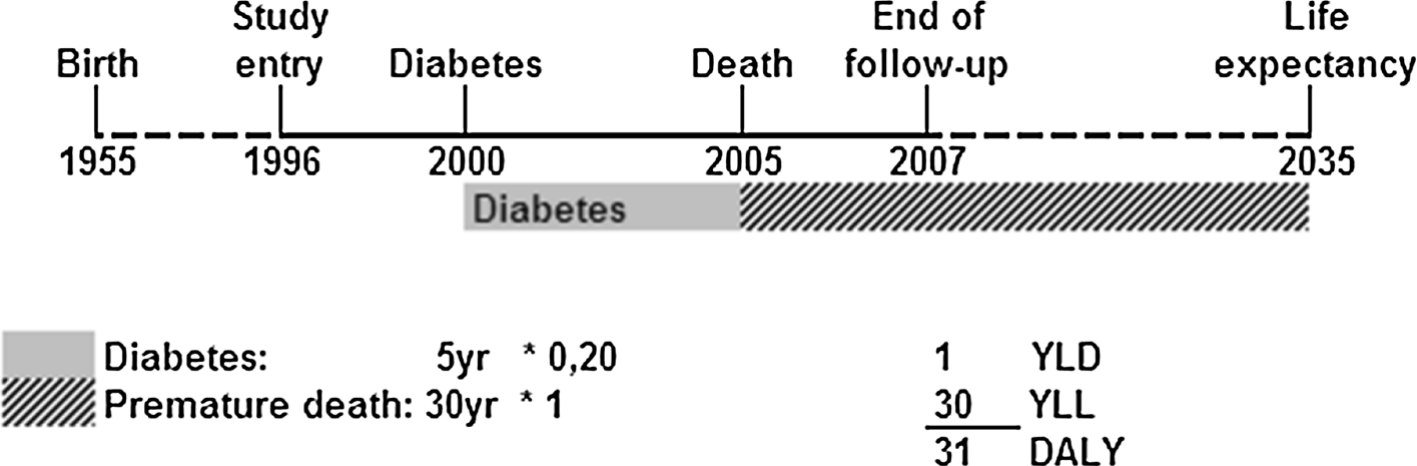
Example of DALY calculation (Reproduced from Struijk et al. [17])

DALYs, i.e. the sum of the Years Lost due to Disability (YLD) and the Years of Life Lost due to premature mortality (YLL) [26], were calculated for each individual in the cohort as previously described [17]. The YLL were computed as the number of years that death occurred earlier than expected. The expected number of life years (life expectancy) are the remaining years that a person of a certain age is expected to live on average defined at the time of death, loss of follow-up or end of follow-up [17]. The expected life expectancy was obtained from Statistics Netherlands that provides age, sex and calendar year specific life expectancies based on mortality rates [27]. The YLD are calculated by the number of years a person lives with a disability multiplied by a disability weight reflecting the severity of that disability. The disability weights were derived from the Dutch Disability Weight study [28]. The disability weights can range between zero (no burden, i.e. full health) and one (death) (Supplemental Table 1). The years lived with a chronic disease are calculated from the disease onset until death or until the end of life expectancy. One DALY represents the loss of one year in full health. For example, a person who lived five years with diabetes (disability weight 0.20) and died 30 years before his life expectancy obtains one YLD and 30 YLL which equals 31 healthy years of life lost (31 DALYs) (Fig. 1). Total DALYs were computed, as well as DALYs for specific diseases: CHD, CVD (includes CHD and stroke), and cancer. DALYs from cancer were further separated into alcohol-related (the upper aero-digestive tract, breast, liver, and colorectal cancer) and non-alcohol related cancer. DALYs for participants that suffered from both alcohol- and non-alcohol-related cancers were included in the alcohol-related cancer DALYs. Potentially alcoholism-related disorders were not included, since several of these disorders were not chronic and occurred in less than 1% of our cohort. However, to explore the influence of these disorders on the association between alcohol consumption and total disease burden, we conducted a sensitivity analysis as a worst-case scenario. For this analysis, we assumed that all of these disorders were chronic. Thus, in this analysis, disorders with a transient nature such as delirium and psychosis were assumed to be present from the date of first diagnosis onwards. The following potentially alcoholism-related disorders were included in these analyses and obtained from the hospital discharge diagnosis database: alcohol dependence, delirium, liver cirrhosis, psychosis and mental disorders.

### Statistical analysis

Due to the distribution of the DALYs, i.e. a peak at 0 and a normal distribution in participants with DALYs > 0, we used a two-part model to estimate the association of alcohol intake with DALYs as dependent variable [29]. This two-part model combines the estimation of the probability of having DALYs using logistic regression with the estimation of the number of DALYs among participants with DALYs > 0 using linear regression. Alcohol intake was analyzed categorically, using light drinkers (0–5 g/day) as the reference. Confidence intervals were constructed with bootstrapping (500 samples). A quadratic P for trend was investigated by applying a regression with quadratic contrast coefficients to DALY estimates of each of the categories, excluding lifetime abstainers and former drinkers, in all bootstrapping samples. The P value was the percentage of positive (or negative, depending on the direction of the association) regression coefficients of the bootstrap times two because of two sided testing.

The analyses were adjusted for age, sex, physical activity level, education level, smoking status and intensity and energy intake (kcal/day). In a second model, BMI (continuous) and diet (continuous mMDS score) were included as covariates. We investigated whether associations differed across strata of age and sex by including an interaction term in the logistic and linear model and by stratifying the analyses by sex and age (>50/≤50 years). To exclude possible residual confounding by smoking, the analyses were repeated among never-smokers only. To explore the influence of alcoholism-related disorders, the analyses were repeated while including these disorders in the DALY calculation. Statistical analyses were conducted using SAS 9.2 (SAS Institute, Cary, US) and SPSS 14.0 (Chicago, IL, USA).

## Results

The percentage of men was higher for moderate (42%) and heavy (31%) drinkers than light drinkers (14%). Furthermore, moderate and heavy drinkers were higher educated, had a slightly lower BMI, smoked more often and had a higher energy intake than light drinkers (Table 1). After a median follow-up of 12.4 years, 6647 disease incidences and 1482 deaths were documented. During follow-up, 68,225 healthy years of life (DALYs) were lost.

**Table 1:** Characteristics of 33,066 EPIC-NL participants at recruitment according to alcohol consumption categories

	Alcohol intake categories					
	Lifetime abstainer	Former drinker	Light 0 to <5 g/day	Moderate	Heavy	Excessive
				Women 5 to <15 g/day	Women 15 to <30 g/day	Women ≥30 g/day
				Men 5 to <30 g/day	Men 30 to <60 g/day	Men ≥ 60 g/day
Total, N	2267	363	13,831	9983	4589	2033
Women (%)	79	52	86	58	69	83
Age at recruitment (years)	43 ± 13	46 ± 11	50 ± 12	47 ± 12	50 ± 10	52 ± 8
BMI (kg/m^2^)	26.1 ± 4.6	25.5 ± 4.2	25.9 ± 4.2	25.3 ± 3.6	25.2 ± 3.5	25.2 ± 3.7
Waist circumference (cm)	85 ± 12	87 ± 13	84 ± 11	86 ± 11	85 ± 11	85 ± 11
High education (%)	10	18	14	28	29	29
Physically active (%)	39	44	42	45	41	38
Current smokers (%)	27	36	24	32	39	53
Energy intake (kcal/day)	2019 ± 611	2181 ± 700	1922 ± 541	2203 ± 644	2513 ± 623	2164 ± 615
Alcohol intake (g/day) (median IQR)	0 (0)	0 (0)	1 (2)	10 (7)	24 (12)	41 (26)
mMDS	3.8 ± 1.5	4.1 ± 1.6	3.9 ± 1.5	4.1 ± 1.5	4.1 ± 1.5	4.0 ± 1.5
Women, N	1784	187	11,888	5747	3187	1691
Age at recruitment (years)	43 ± 13	46 ± 12	52 ± 12	50 ± 11	52 ± 9	53 ± 8
BMI (kg/m^2^)	26.2 (4.8)	24.9 (4.3)	25.9 (4.3)	25.0 (3.7)	24.9 (3.4)	25.1 (3.7)
Waist circumference (cm)	84 (12)	82 (11)	83 (11)	81 (10)	81 (9)	83 (10)
High education (%)	8	21	13	25	28	29
Physically 7active (%)	37	41	42	44	41	37
Current smokers (%)	27	32	22	28	35	50
Energy intake (kcal/day)	1906 ± 530	1844 ± 480	1828 ± 459	1906 ± 449	1916 ± 441	2002 ± 450
Alcohol intake, g/day (median IQR)	0 (0)	0 (0)	1 (2)	9 (5)	21 (6)	38 (14)
mMDS	3.8 ± 1.4	4.2 ± 1.6	3.9 ± 1.5	4.1 ± 1.5	4.1 ± 1.5	4.1 ± 1.5
Men, N	483	176	1943	4236	1402	342
Age at recruitment (years)	41 ± 12	46 ± 10	42 ± 12	42 ± 11	45 ± 10	46 ± 9
BMI (kg/m^2^)	25.8 (3.6)	26.2 (4.0)	25.6 (3.6)	25.6 (3.3)	26 (3.4)	25.9 (3.9)
Waist circumference (cm)	92 (11)	94 (12)	91 (11)	92 (10)	94 (10)	95 (11)
High education (%)	15	15	20	32	31	29
Physically active (%)	45	48	47	47	41	39
Current smokers (%)	28	41	32	36	49	65
Energy intake (kcal/day)	2439 ± 701	2538 ± 720	2495 ± 642	2606 ± 648	2692 ± 641	2964 ± 695
Alcohol intake (g/day) (median IQR)	0 (0)	0 (0)	2 (3)	14 (11)	39 (13)	75 (24)
mMDS	3.9 ± 1.6	4.0 ± 1.6	3.9 ± 1.4	4.0 ± 1.5	4.0 ± 1.5	3.7 ± 1.4

*mMDS* modified Mediterranean Diet Score

In crude analyses, moderate alcohol consumption was associated with a significantly lower total disease burden (mean DALYs −0.42; 95% CI −0.55; −0.28) compared with light drinkers (Table 2). Adjusting these analyses for confounders did not substantially alter the results for moderate drinkers. Moderate drinkers still had a significantly lower total disease burden (mean DALYs −0.27; 95% CI −0.43; −0.11). This corresponds to living approximately 3 months longer in good health for moderate drinkers. When separating DALY’s due to CVD and cancer, moderate alcohol consumption was only associated with a lower disease burden from cardiovascular causes (mean DALYs −0.18; 95% CI −0.29; −0.06), but not from cancer (mean DALYs −0.05; 95% CI −0.16; 0.06) (Table 2). In crude analysis, substantial drinking was not associated with total disease burden (mean DALYs 0.02; 95% CI −0.13; 0.18). After multivariable adjustment, substantial drinking was associated with a lower total disease burden (mean DALYs −0.18; 95% CI −0.35; 0.00) (Table 2). Exchanging BMI for waist circumference in the model did not substantially alter the results (data not shown).

**Table 2:** Mean (±SE) DALYs lost and regression coefficients (95% CI) of different alcohol consumption categories with DALYs in 33,066 EPIC-NL participants

	Alcohol intake categories					
	Lifetime abstainer	Former drinker	Light 0– <5 g/day	Moderate	Substantial	Quadratic *P* for trend
				Women 5– <15 g/day	Women ≥15 g/day	
				Men 5– <30 g/day	Men ≥30 g/day	
N	2267	363	13,831	9983	6622	
*DALY total*	4534	1050	30,428	17,969	14,568	
Mean DALY (SD)	2.0 (5.2)	2.9 (6.9)	2.2 (5.2)	1.8 (4.8)	2.2 (5.4)	
Crude	−0.22 (−0.45; −0.00)	0.73 (0.06; 1.44)	Reference	−0.42 (−0.55; −0.28)	0.02 (−0.13; 0.18)	<0.01
Adjusted 1^a^	0.22 (−0.08; 0.52)	0.74 (−0.01; 1.52)	Reference	−0.31 (−0.48; −0.15)	−0.22 (−0.40; −0.04)	0.008
Adjusted 2^b^	0.16 (−0.13; 0.46)	0.81 (0.03; 1.59)	Reference	−0.27 (−0.43; −0.11)	−0.18 (−0.35; −0.00)	0.02
*DALY coronary heart disease*						
Mean DALY						
Crude	0.00 (−0.08; 0.09)	0.28 (−0.01; 0.56)	Reference	−0.03 (−0.09; 0.02)	−0.01 (−0.08; 0.04)	0.33
Adjusted 1^a^	0.06 (−0.11; 0.23)	0.07 (−0.27; 0.41)	Reference	−0.15 (−0.24; −0.06)	−0.18 (−0.28; −0.09)	0.14
Adjusted 2^b^	0.04 (−0.12; 0.22)	0.08 (−0.26; 0.41)	Reference	−0.14 (−0.23; −0.05)	−0.17 (−0.27; −0.07)	0.18
*DALY cardiovascular disease (coronary heart disease* + *stroke)*						
Mean DALY						
Crude	−0.02 (−0.14; 0.10)	0.31 (−0.04; 0.69)	Reference	−0.09 (−0.16; −0.02)	−0.01 (−0.09; 0.07)	0.01
Adjusted 1^a^	0.08 (−0.14; 0.32)	0.17 (−0.29; 0.66)	Reference	−0.19 (−0.30; −0.07)	−0.19 (−0.31; −0.05)	0.08
Adjusted 2^b^	0.06 (−0.15; 0.29)	0.19 (−0.28; 0.66)	Reference	−0.18 (−0.29; −0.06)	−0.18 (−0.29; −0.04)	0.10
*DALY cancer*						
Mean DALY						
Crude	−0.25 (−0.39; −0.11)	0.13 (−0.30; 0.60)	Reference	−0.14 (−0.23; −0.06)	0.07 (−0.04; 0.17)	<0.01
Adjusted 1^a^	−0.09 (−0.28; 0.12)	0.21 (−0.33; 0.78)	Reference	−0.06 (−0.17; 0.05)	−0.05 (−0.17; 0.06)	0.51
Adjusted 2^b^	−0.10 (−0.29; 0.11)	0.22 (−0.33; 0.80)	Reference	−0.05 (−0.16; 0.06)	−0.04 (−0.17; 0.07)	0.54

^a^Adjusted model 1 is adjusted for age, sex, physical activity, education, energy intake, smoking status and intensity

^b^ Adjusted model 2 = model 1 + bmi + mMDS

When subdividing substantial drinkers into heavy and excessive drinkers, the association with lower total disease burden attenuated at higher levels of intake (Table 3). Excessive alcohol consumption was not associated with total DALYs (mean DALYs −0.02; 95% CI −0.28; 0.27) adjusted for confounders. Separating these analyses for DALYs due to CVD and cancer showed that excessive compared to light alcohol consumption remained associated with a lower CVD disease burden (mean DALYs −0.21; −0.40; −0.01), but with a slightly higher cancer disease burden (mean DALYs 0.08; 95% CI −0.11; 0.29). Finally, separating DALYs from cancer into alcohol-related and non-alcohol related cancer showed that excessive alcohol consumption was significantly associated with a higher disease burden from alcohol-related cancer (mean DALYs 0.11; 95% CI 0.01; 0.21), but not associated with disease burden from non-alcohol related cancer (mean DALYs −0.04; 95% CI −0.20; 0.14).

**Table 3:** Mean (±SE) DALYs lost and regression coefficients^a^ (95% CI) of different alcohol consumption categories with DALYs in 33,066 EPIC-NL participants, separating substantial drinking categories

	Alcohol intake categories						
	Lifetime abstainer	Former drinker	Light 0 to <5 g/day	Moderate	Heavy	Excessive	Quadratic *P* for trend
				Women 5 to <15 g/day	Women 15 to <30 g/day	Women ≥30 g/day	
				Men 5 to <30 g/day	Men 30 to <60 g/day	Men ≥60 g/day	
N	2267	363	13,831	9983	4589	2033	
*DALY total*							
Crude	−0.22 (−0.45; −0.00)	0.73 (0.06; 1.44)	Reference	−0.42 (−0.55; −0.28)	−0.16 (−0.33; −0.00)	0.44 (0.17; 0.73)	<0.01
Adjusted	0.16 (−0.13; 0.46)	0.81 (0.03; 1.59)	Reference	−0.27 (−0.43; −0.11)	−0.26 (−0.45; −0.07)	−0.02 (−0.28; 0.27)	0.004
*DALY coronary heart disease*							
Crude	0.00 (−0.08; 0.09)	0.28 (−0.01; 0.56)	Reference	−0.03 (−0.09; 0.02)	−0.00 (−0.07; 0.06)	−0.04 (−0.14; 0.06)	0.95
Adjusted	0.04 (−0.12; 0.22)	0.08 (−0.26; 0.41)	Reference	−0.14 (−0.23; −0.05)	−0.15 (−0.25; −0.03)	−0.23 (−0.38; −0.08)	0.62
*DALY cardiovascular disease (coronary heart disease* + *stroke)*							
Crude	−0.02 (−0.14; 0.10)	0.31 (−0.04; 0.69)	Reference	−0.09 (−0.16; −0.02)	−0.02 (−0.11; 0.08)	0.01 (−0.13; 0.17)	0.20
Adjusted	0.06 (−0.15; 0.29)	0.19 (−0.28; 0.66)	Reference	−0.17 (−0.29; −0.06)	−0.16 (−0.30; −0.01)	−0.21 (−0.40; −0.01)	0.36
*DALY cancer*							
Crude	−0.25 (−0.39; −0.11)	0.13 (−0.30; 0.60)	Reference	−0.14 (−0.23; −0.06)	−0.06 (−0.18; 0.04)	0.35 (0.15; 0.55)	<0.01
Adjusted	−0.10 (−0.29; 0.11)	0.22 (−0.33; 0.80)	Reference	−0.06 (−0.16; 0.06)	−0.10 (−0.25; 0.01)	0.08 (−0.11; 0.29)	0.03
*DALY alcohol*-*related cancer*							
Crude	−0.14 (−0.23; −0.03)	0.02 (−0.26; 0.33)	Reference	−0.08 (−0.13; −0.03)	−0.03 (−0.11; 0.03)	0.18 (0.05; 0.31)	<0.01
Adjusted	−0.08 (−0.17; 0.02)	0.10 (−0.19; 0.42)	Reference	0.01 (−0.05; 0.06)	0.00 (−0.07; 0.06)	0.11 (0.01; 0.21)	0.10
*DALY non*-*alcohol*-*related cancer*							
Crude	−0.11 (−0.21; 0.01)	0.11 (−0.20; 0.50)	Reference	−0.06 (−0.13; 0.01)	−0.03 (−0.12; 0.06)	0.17 (0.02; 0.34)	0.008
Adjusted	0.01 (−0.21; 0.22)	0.09 (−0.34; 0.61)	Reference	−0.06 (−0.16; 0.05)	−0.13 (−0.27; −0.02)	−0.04 (−0.20; 0.14)	0.15

^a^Adjusted model is adjusted for age, sex, physical activity, education, energy intake, smoking status and intensity, BMI and mMDS; alcohol-related = cancer of the upper aero-digestive tract, breast, liver, and colorectum

In a sensitivity analysis including alcoholism-related disorders, the association between moderate alcohol consumption and lower total disease burden did not materially change (mean DALYs −0.26; 95% CI −0.42; −0.10). Substantial drinking still was not associated with total disease burden (mean DALYs −0.12; 95% CI −0.31; 0.07), but the estimate was substantially attenuated. Excessive drinking was related with a higher total disease burden, albeit statistically non-significant (mean DALYs 0.12; 95% CI −0.17; 0.45). Substantial drinking was associated with a significantly higher disease burden for alcoholism-related disorders (mean DALYs 0.10; 95% CI 0.06; 31.4).

Stratified by age (Table 4), moderate alcohol consumption was more strongly associated with a lower total disease burden among older participants (≥50 years; mean DALYs −0.32; 95% CI −0.53; −0.10), than among younger participants (<50 years; mean DALYs −0.14; 95% CI −0.38; 0.06), although the interaction between age and alcohol was not statistically significant (*p* > 0.14). Results did not differ between men and women (p_interaction_ > 0.40). Stratifying by age and sex showed that the lower total disease burden with moderate alcohol consumption was present among older women only, but not among younger women (mean DALYs −0.08; 95% CI −0.43; 0.35).

**Table 4:** Mean (± SE) DALYs lost and regression coefficients (95% CI)^a^ of different alcohol consumption categories with DALYs in 33,066 EPIC-NL participants of the EPIC-NL cohort, stratified by age group and sex

	Alcohol intake categories					
	Lifetime abstainer	Former drinker	Light 0– <5 g/day	Moderate	Substantial	Quadratic *p* for trend
				Women 5– <15 g/day	Women ≥15 g/day	
				Men 5– <30 g/day	Men ≥30 g/day	
*DALY total*						
<50 years	0.02 (−0.31; 0.35)	1.33 (0.41; 2.36)	Reference	−0.14 (−0.38; 0.06)	−0.04 (−0.31; 0.21)	0.22
≥50 years	0.19 (−0.23; 0.59)	0.02 (−0.94; 1.09)	Reference	−0.32 (−0.53; −0.10)	−0.26 (−0.49; −0.03)	0.10
*DALY total*						
men	−0.15 (−0.68; 0.46)	0.78 (−0.19; 1.85)	Reference	−0.31 (−0.67; 0.02)	−0.18 (−0.59; 0.18)	0.05
women	0.22 (−0.10; 0.54)	0.56 (−0.54; 1.72)	Reference	−0.25 (−0.42; −0.06)	−0.18 (−0.35; 0.01)	0.06
*DALY total*						
<50 years, men	−0.11 (−0.77; 0.59)	1.30 (0.04; 2.62)	Reference	−0.26 (−0.68; 0.11)	−0.32 (−0.77; 0.11)	0.56
≥50 years, men	−0.20 (−1.20; 1.09)	0.12 (−1.38; 1.66)	Reference	−0.28 (−0.89; 0.28)	0.15 (−0.55; 0.78)	0.35
<50 years, women	0.06 (−0.35; 0.54)	1.09 (−0.19; 3.47)	Reference	−0.08 (−0.43; 0.35)	0.13 (−0.25; 0.55)	0.35
≥50 years, women	0.30 (−0.13; 0.73)	0.11 (−1.21; 1.50)	Reference	−0.28 (−0.47; −0.05)	−0.28 (−0.51; −0.04)	0.22

^a^Adjusted for age, sex, physical activity, education, energy intake, smoking status and intensity, BMI and mMDS

Repeating the analyses among never-smokers only (2700 men and 10,080 women), similar results were obtained for moderate drinkers (mean DALYs −0.24; 95% CI −0.48; −0.04), but no significant associations for other alcohol consumption categories.

## Discussion

This study investigated the relation of alcohol consumption with chronic disease burden, thus summarizing opposing associations of alcohol with different diseases. We found that moderate alcohol consumption (5–14.9 g/day for women, 5–29.9 g/day for men) was associated with a lower total disease burden compared to light consumption (0–4.9 g/day). This association was mainly observed among women and older participants and driven by a lower disease burden due to CVD. No association was observed among younger women. Substantial alcohol consumption was not associated with burden from chronic diseases. However, excessive alcohol consumption was associated with higher disease burden from alcohol-related cancer.

The main strength of our study is its prospective design with a summary health measure as outcome. However, several limitations need to be addressed. Left and right truncation of the cohort may have led to an underestimation of the true association between alcohol consumption and chronic disease burden [17]. Participants who were still alive at the end of follow-up were assumed to stay in the same state of health until their expected age of death. In reality, part of the participants who were still disease-free at the end of follow-up will develop disease or die before the expected age. This is not accounted for in the current analysis. For those living with a disease at the end of follow-up, DALYs may be underestimated as well, since their life expectancy is assumed to be similar to that of a healthy person, while they are likely to die earlier. Due to the relatively healthy cohort and exclusion of participants with prevalent diseases at baseline, many participants (79%) were still disease-free at the end of follow-up. Although we included several potentially alcoholism-related disorders in a sensitivity analysis, underestimation of the association for substantial consumption may still be present due to the inability to include Alzheimer’s disease, depression and alcohol-related ‘diseases’ such as injuries for which disability weights are not available. Furthermore, the number of some of the included diseases may be underestimated because these were based on hospital discharge diagnoses, which includes only severe cases needing hospitalization. Finally, disease burden was based on an observation time of 12 years. So, the total number of DALYs will be underestimated. Due to the non-linear relation, it is not clear how these relate to alcohol consumption and thus to predict its effect on the observed relations. Another limitation is the self-reported alcohol intake data, although alcohol consumption from the FFQ correlated well with the result of twelve monthly 24-h recalls [19, 20]. Also, we had relatively few excessive drinkers in our cohort and we could not account for drinking patterns like binge drinking. However, binge drinking does not frequently occur among middle-aged or older persons (>55 years) [30]. It is therefore unlikely that this has influenced the results in this cohort of predominantly middle-aged and older women to a large extent. Furthermore, we studied relations with disease burden; societal consequences of alcohol consumption were not included. Finally, we cannot rule out the possibility of residual confounding, as in any observational study.

So far DALYs were mainly used in GBD studies, which aim to define the health status in (different parts of) the world and over time, using population data and modeling techniques. According to the GBD Study of 2010, alcohol use accounted for up to 5% of the global disease burden [11], ranging widely according to regions. These estimates cannot be compared to our estimate. We find lower chronic disease burden for moderate drinkers and higher burden for substantial drinkers. The relative distribution of drinking habits in the population will determine the overall association. We further focused on chronic diseases in an adult population, while a younger populations with different drinking patterns and the inclusion of accidents and liver cirrhosis may affect population estimates. In the GBD studies the disease burden of alcohol is highest for populations with a high proportion of heavy drinkers and thus a high contribution of alcohol related diseases to disease burden. Our study consists predominantly of middle-aged or older women and included relatively few heavy drinkers, leading to a low contribution of alcohol related conditions to disease burden.

The net lower total disease burden of approximately 3 months among moderate drinkers compared with light drinkers seems to be in line with evidence from cohort studies showing a reduced risk of total mortality with moderate alcohol consumption [5]. Two prospective cohort studies related alcohol consumption to life expectancy [31, 32]. Streppel et al. [32] showed that consuming ≤20 g alcohol/day compared with no alcohol consumption was associated with a 5-year longer life expectancy at age 50. The effect size of this study was larger than we observed. This may partly be due to the fact that the reference category included former drinkers, while we excluded them from the reference as they are likely to be sick-quitters [33]. Li et al. [31] showed that heavy alcohol consumption (>4 drinks/day for men, >1 drinks/day for women) compared with light consumption was associated with a reduction of 4 years in life expectancy. Based on a life table approach, Klijs et al. [34] also estimated that heavy drinking was associated with approximately 2 years living with disability. We found no relation with chronic disease burden for substantial drinkers, but excessive drinking was associated with alcohol related diseases.

In our study, disease burden associated with alcohol consumption differed by age. The lower chronic disease burden with moderate alcohol consumption was most pronounced among participants aged 50 years and over, and not observed among women aged <50 years. These differences are mainly due to different diseases attributing to disease burden. Since CVD account for the majority of disease burden among older adults, the net association is mainly driven by the association of alcohol consumption and CVD. Women under age 50 years more frequently suffer from breast cancer than CVD, resulting in a null net association for alcohol consumption. In our study cancer indeed accounted for approximately 60% of deaths among women <50 years, while this was lower in the other age/sex groups.

In conclusion, our results provide an estimate of the contribution of alcohol consumption to disease burden over a period of 12 years. The lower chronic disease burden with moderate alcohol consumption supports the current guidelines allowing moderate alcohol consumption up to 1 drink/day for women and up to 2 drinks/day for men and discouraging higher levels of alcohol consumption [1–4]. However, our results suggest that the recommendations for moderate alcohol consumption mainly apply to middle-aged or older populations.

## Electronic supplementary material

Below is the link to the electronic supplementary material.

Supplementary material 1 (DOC 70 kb)
