# Supplementary material for: Moderate alcohol consumption is associated with lower chronic disease burden expressed in disability-adjusted life years: a prospective cohort study
Source: Eur J Epidemiol. 2017 Apr 13;32(4):317–26. doi: 10.1007/s10654-017-0247-x (PMC5437140; doi:10.1007/s10654-017-0247-x)
Supplement: Supplementary file 1 — Supplementary material 1 (DOC 70 kb) [file 10654_2017_247_MOESM1_ESM.doc]

Supplemental table 1 Disability weights for disabilities and different cancer types

| **Diseases** | **Disability weight** | | **Source** |
| --- | --- | --- | --- |
| Coronary heart disease | | 0.29 | Dutch disability weight |
| Stroke | | 0.61 | Dutch disability weight |
| Diabetes mellitus | | 0.20 | Dutch disability weight |
| Chronic Obstructive Pulmonary disease | | 0.31 | Dutch disability weight |
| Asthma | | 0.08 | Dutch disability weight |
| Parkinson´s disease | | 0.68 | Dutch disability weight |
| Rheumatoid arthritis | | 0.53 | Dutch disability weight |
| Osteoarthritis | | 0.19 | Dutch disability weight |
| Inflammatory bowel disease | | 0.20 | Dutch disability weight |
|  | |  |  |
| **Cancer types** | |  |  |
| **Digestive** | |  |  |
| Stomach | | 0.59 | Dutch disability weight |
| Oesophagus | | 0.53 | Dutch disability weight |
| Colon | | 0.30 | Dutch disability weight |
| Pancreas | | 0.53 | Based on Dutch disability weight for oesophagus cancer |
| Unspecified parts of billary tract | | 0.53 | Based on Dutch disability weight for oesophagus cancer |
| Galbladder | | 0.53 | Based on Dutch disability weight for oesophagus cancer |
| Liver and intrahepatic bile ducts | | 0.53 | Based on Dutch disability weight for oesophagus cancer |
| Rectum | | 0.30 | Based on Dutch disability weight for colon cancer |
| Rectosigmoid junction | | 0.30 | Based on Dutch disability weight for colon cancer |
| Small intestine | | 0.30 | Based on Dutch disability weight for colon cancer |
| Anus and anal canal | | 0.30 | Based on Dutch disability weight for colon cancer |
| Other and ill-defined digestive organs | | 0.30 | Based on Dutch disability weight for colon cancer |
| **Respiratory, intrathoracic** | |  |  |
| Bronchus and lung | | 0.54 | Dutch disability weight |
| Heart, mediastinum and pleura | | 0.54 | Based on Dutch disability weight for lung cancer |
| Accessory sinuses | | 0.54 | Based on Dutch disability weight for lung cancer |
| Larynx | | 0.54 | Based on Dutch disability weight for lung cancer |
| **Male genital organs** | |  |  |
| Prostate | | 0.26 | Dutch disability weight |
| **Breast** | |  |  |
| Breast | | 0.26 | Dutch disability weight |
| **Hematopoetic and reticuloendothelial** | |  |  |
| Hematopoetic and reticuloendothelial | | 0.24 | Dutch disability weight |
| **Female genital organs** | |  |  |
| Cervix uteri | | 0.12 | Dutch disability weight |
| Vulva | | 0.12 | Based on Dutch disability weight for cervix uteri cancer |
| Ovary | | 0.30 | Based on Dutch disability weight for colon cancer |
| Corpus uteri | | 0.12 | Based on Dutch disability weight for cervix uteri cancer |
| Unspecified female genital organs | | 0.30 | Based on Dutch disability weight for colon cancer |
| **Skin** | |  |  |
| Skin | | 0.08 | Dutch disability weight |
| **Brain, central nervous system** | |  |  |
| Brain | | 0.54 | Based on Australian disability weight for brain cancer |
| Spinal cord, cranial nerves, other parts of central nervous system | | 0.54 | Based on Australian disability weight for brain cancer |
| **Urinary tract** | |  |  |
| Kidney, except renal pelvis | | 0.26 | Based on Dutch disability weight for prostate cancer |
| Bladder | | 0.26 | Based on Dutch disability weight for prostate cancer |
| Ureter | | 0.26 | Based on Dutch disability weight for prostate cancer |
| **Mesothelial, soft tissue** | |  |  |
| Retroperitoneum and peritoneum | | 0.30 | Based on Dutch disability weight for colon cancer |
| Other connective and soft tissue | | 0.30 | Based on Dutch disability weight for colon cancer |
| **Thyroid endocrine gland** | |  |  |
| Thyroid gland | | 0.20 | Based on Dutch disability weight for thyroid cancer |
| **Lip, oral cavity, pharynx** | |  |  |
| Pyriform sinus | | 0.53 | Based on Dutch disability weight for oesophagus cancer |
| Tonsil | | 0.53 | Based on Dutch disability weight for oesophagus cancer |
| Base of tongue | | 0.53 | Based on Dutch disability weight for oesophagus cancer |
| Floor of mouth | | 0.53 | Based on Dutch disability weight for oesophagus cancer |
| Oropharynx | | 0.53 | Based on Dutch disability weight for oesophagus cancer |
| Nasopharynx | | 0.53 | Based on Dutch disability weight for oesophagus cancer |
| Gum | | 0.53 | Based on Dutch disability weight for oesophagus cancer |
| Other unspecified parts of tongue | | 0.53 | Based on Dutch disability weight for oesophagus cancer |
| Other and ill-defined sites in lip, oral cavity and pharynx | | 0.53 | Based on Dutch disability weight for oesophagus cancer |
| **Bone and articular cartilage** | |  |  |
| Bone and articular cartilage of limbs | | 0.30 | Based on Australian disability weight for bone cancer |

Obtained from Melse et al. (21)
